# Beneficial Features of Biochar and Arbuscular Mycorrhiza for Improving Spinach Plant Growth, Root Morphological Traits, Physiological Properties, and Soil Enzymatic Activities

**DOI:** 10.3390/jof7070571

**Published:** 2021-07-17

**Authors:** Dilfuza Jabborova, Kannepalli Annapurna, Sangeeta Paul, Sudhir Kumar, Hosam A. Saad, Said Desouky, Mohamed F. M. Ibrahim, Amr Elkelish

**Affiliations:** 1 Institute of Genetics and Plant Experimental Biology, Uzbekistan Academy of Sciences, Tashkent Region, Kibray 111208, Uzbekistan; 2 Division of Microbiology, ICAR-Indian Agricultural Research Institute, Pusa, New Delhi 110012, India; sangeeta_paul2003@yahoo.co.in; 3 Division of Plant Physiology, ICAR-Indian Agricultural Research Institute, New Delhi 110012, India; sudhir_physiol@iari.res.in; 4 Department of Chemistry, College of Science, Taif University, P.O. Box 11099, Taif 21944, Saudi Arabia; h.saad@tu.edu.sa; 5 Department of Botany and Microbiology, Faculty of Science, Al-azhar University, Cairo 11884, Egypt; said.desouky@azhar.edu.eg; 6 Department of Agricultural Botany, Faculty of Agriculture, Ain Shams University, Cairo 11566, Egypt; Ibrahim_mfm@agr.asu.edu.eg; 7 Botany Department, Faculty of Science, Suez Canal University, Ismailia 41522, Egypt; amr.elkelish@science.suez.edu.eg

**Keywords:** spinach, biochar, AMF, plant growth, root morphological traits, chlorophyll content, soil enzymes and microbial biomass

## Abstract

Biochar and arbuscular mycorrhizal fungi (AMF) can promote plant growth, improve soil properties, and maintain microbial activity. The effects of biochar and AMF on plant growth, root morphological traits, physiological properties, and soil enzymatic activities were studied in spinach (*Spinacia oleracea* L.). A pot experiment was conducted to evaluate the effect of biochar and AMF on the growth of spinach. Four treatments, a T1 control (soil without biochar), T2 biochar alone, T3 AMF alone, and T4 biochar and AMF together, were arranged in a randomized complete block design with five replications. The biochar alone had a positive effect on the growth of spinach, root morphological traits, physiological properties, and soil enzymatic activities. It significantly increased the plant growth parameters, such as the shoot length, leaf number, leaf length, leaf width, shoot fresh weight, and shoot dry weight. The root morphological traits, plant physiological attributes, and soil enzymatic activities were significantly enhanced with the biochar alone compared with the control. However, the combination of biochar and AMF had a greater impact on the increase in plant growth, root morphological traits, physiological properties, and soil enzymatic activities compared with the other treatments. The results suggested that the combined biochar and AMF led to the highest levels of spinach plant growth, microbial biomass, and soil enzymatic activity.

## 1. Introduction

Biochar is the carbon-rich material taken by pyrolysis using various biomasses. Biochar plays an important role in decreasing global warming in the world and reducing atmospheric CO_2_ concentrations, as well as improving soil used in agriculture [1,2,3,4,5,6]. Biochar application has been noted to increase the activity of microbes in the soil and improve the physical and chemical properties of the soil [7,8,9,10,11,12,13]. Ścisłowska et al. [14] observed that biochar treatments improved the quality and productivity of soils. Laird et al. [15] reported that biochar treatments significantly increased the water-holding capacity, cation exchange capacity, and specific surface area of soils. Rice husk biochar significantly increased the organic matter in soil by 52.94% compared with the control [16]. Numerous reports have indicated that biochar alone increased the activities of dehydrogenase, alkaline phosphatase, acid phosphatase, acid phosphomonoesterase, alkaline phosphomonoesterase, protease, chymotrypsin, trypsin, phosphohydrolase, lipase-esterase, and esterase enzymes [17,18,19].

Biochar also plays an important role in soil nutrient availability and adsorption. Biochar promotes soil nutrients, such as nitrogen, potassium, calcium, magnesium, sodium, and total carbon [20,21]. Jabborova et al. [18] reported that the addition of biochar to soil significantly increased the content of nitrogen, phophorus, and potassium compared to the control.

Biochar had a positive effect on the growth, development, yield, and nutrient content of different plants. Several reports have indicated that biochar increased the seed germination, plant growth, and yield of soybeans [8,22,23,24,25]. The germination rate was highest when biochar was used compared to the control [20]. Concentrations of calcium and magnesium in maize leaves were significantly higher with a high biochar application rate than with the control [5]. Biochar application improved the root and shoot biomass of *Plantago lanceolata* [26]. Rice straw biochar significantly increased the plant height, number of bolls per plant, average boll weight, and seed cotton yield compared to the control [16]. Carter et al. [27] reported that rice husk biochar application increased the final biomass, root biomass, plant height, and number of leaves of lettuce (*Lactuca sativa*) and cabbage (*Brassica chinensis*) in comparison with plants that had not received biochar.

Biochar has a positive impact on plant physiological and biochemical properties. Several studies have shown that biochar application increased the plant photosynthesis, chlorophyll content, and transpiration rate [19,28,29]. Biochar amendment significantly increased the photosynthetic rate of okra (*Abelmoschus esculentus* L.) [25].

Arbuscular mycorrhizal fungi (AMF) is a major component of the rhizosphere microflora in natural ecosystems, and plays a significant role in ecosystems through nutrient cycling [30,31]. Mycorrhiza are microbes that promote plant growth and play an important role in enhancing plant nutrition [32,33,34]. AMF have a symbiotic relationship with plants. Inoculating crop roots with mycorrhiza improves the uptake of nutrients, such as nitrogen, potassium, phosphorus, calcium, and magnesium [35,36,37,38]. The plants inoculated with mycorrhiza had increased chlorophyll and carotenoid content and increased antioxidant enzymes, such as superoxide, dismutase, catalase, peroxidase, and ascorbate peroxidase [39]. The use of mycorrhiza helped to improve the branching of the plant root system and the growth and productivity of several field crops [40,41,42]. Several researchers have reported that inoculation with both mycorrhiza and plant growth‒promoting rhizobacteria (PGPR) could be beneficial for agriculture [43,44]. Biochar and mycorrhiza have proved useful for enhancing plant growth and yield and reducing the intensity of disease. IIA [45] reported that dual inoculation with AMF and biochar significantly increased plant growth and the phosphorus content of maize.

Spinach (*Spinacia oleracea* L.) is high in nutrients that benefit humans, including bioactive compounds, vitamins, and minerals [46]. It is rich in bioactive compounds that contribute to anticancer, antiobesity, hypoglycemic, and hypolipidemic properties [47]. The production of spinach is largely affected by abiotic and biotic stress. In addition to abiotic stress, the combined application of biochar and AMF is also helpful for alleviating the negative impact of biotic stress [48]. However, little information is available on the interactive effect of biochar and AMF on spinach. In this study, we investigated the effect of the combined application of biochar and AMF on spinach growth, root morphological traits, physiological properties, and soil enzymatic activities. We hypothesized that the combined application of biochar and AMF would facilitate beneficial effects on plant growth, plant nutrients, physiological properties, and soil properties.

## 2. Materials and Methods

Soil collected from the Indian Agricultural Research Institute (IARI) was used for the experiment. The biochar used in the study was produced at 400 to 500 °C from a woody biomass (Amazon online shop, New Delhi, India), with a particle size of less than 2 mm. Seeds were obtained from the Division of Vegetable Science, IARI, New Delhi, India, and AMF were obtained from the Division of Microbiology, IARI, New Delhi, India.

### 2.1. Experimental Design

The effect of biochar and AMF on the growth of spinach was studied in pot experiments in a nethouse. All of the experiments were carried out in a randomized block design with five replications. Experimental treatments included a T1 control (soil without biochar), T2 = biochar alone, T3 = AMF alone, and T4 = biochar + AMF. Seeds were sown in plastic pots (20 cm in diameter and 20 cm in depth) containing 5.0 kg of soil. Each pot was watered every three days. At harvest, after 40 days, the shoot length, leaf length, leaf number, leaf width, fresh root weight, fresh shoot weight, dry root weight, and dry shoot weight were measured. Physiological parameters, such as relative water content, net photosynthetic rate, stomatal conductance, and transpiration rate, as well as photosynthetic pigments, were also determined after 40 days.

### 2.2. Measurement of Root Morphological Traits of Spinach

The roots were washed carefully with water. The whole root system was spread out and analyzed using a scanning system (Expression 4990, Epson, CA) with a blue board as a background. Digital images of the root system were analyzed using Win RHIZO software (Régent Instruments, Québec, QC, Canada). The total root length, root surface area, root volume, projected area, and root diameter were evaluated.

### 2.3. Physiological Parameters Measurement

Relative water content (RWC) was measured by the method of Abd El-Gawad, et al. [49]. One hundred mg of fully expanded fresh leaf sample (FW) were placed immediately after sampling in petri plates filled with double distilled water for 4 h at room temperature. The samples were then taken out and blotted dry, and the turgid weight (TW) was recorded. The samples were kept in an oven at 70 °C overnight, and the dry weight (DW) was recorded. Relative water content was calculated as:

RWC (%) = [(FW − DW)/(TW − DW)] × 100

Photosynthetic pigments were determined by the modified method of Hiscox and Israelstam [50]. Fresh leaves were collected in the morning. Fifty mg of fine pieces of fresh leaf sample 2 to 3 mm in size were cut and added to test tubes containing 5 mL of DMSO. Then the test tubes were incubated at 37 °C for 4 h in the dark. The incubation was continued until completely colorless tissue was obtained. The absorbance of the extract was taken at 470 nm, 645 nm, and 663 nm using a spectrophotometer against a DMSO blank. The chlorophyll a (Chl a), chlorophyll b (Chl b), total chlorophyll, and carotenoid contents were determined using the following equations:

Chl a (mg/g) = [12.7(A_663_) − 2.69(A_645_)] × V/W

Chl b (mg/g) = [22.9(A_645_) − 4.68(A_663_)] × V/W

Total Chl (mg/g) = [20.2 (A_645_) + 8.02 (A_663_)] × V/W

Carotenoids (mg/g) = [(1000 × A_470_) − (3.27 × Chl a + 104 × Chl b)] × V/W

where, A = optical density; V = Volume of DMSO (in ml); W = sample weight.

The net photosynthetic rate, stomatal conductance, and transpiration rate were measured using a portable LI-6400XT photosynthesis measurement system between 9:00–11:00 a.m. The fully expanded youngest leaf was used for the measurement. The photosynthetic active radiation (PAR), temperature, and CO_2_ concentration during the measurements were 300 mmol m^−2^ s^−1^, 30 °C, and 400 mmol mol^−1^, respectively.

### 2.4. Analysis of AMF Spores from Soil

The AMF spores were extracted from 10 g soil samples using wet sieving and decanting method. The soil sample was put over a series of soil sieves arranged in descending order of sieve sizes. The clean spores were mesh sieved and washed several times with distilled water before being transferred into water in a clean petri dish. The AMF spores were counted under a stereomicroscope [51].

### 2.5. Analysis of Soil Microbial Biomass Determination

The methods used to measure biomass C were based on those described by Vance et al. [52]. Three of six 17.5 g replicates of each soil sample were fumigated with purified CHCl_3_ for 24 h. After removal of the CHCl_3_, the C was extracted from fumigated and unfumigated samples with 0.5 M of K_2_SO_4_ for 1 h on an end-over-end shaker. Fumigated and unfumigated samples were filtered sequentially through filter paper (Whatman filter grade 42). The obtained supernatant liquid was measured at 280 nm using a spectrophotometer.

### 2.6. Analysis of Soil Enzymes

The alkaline phosphatase activities were assayed with the method by Tabatabai and Bremner [53]. For each soil, two sets of 1 g of soil were placed in conical flasks. One set was used as the control. Then, 0.2 mL of toluene and 4 mL of MUB (modified universal buffer) (pH = 11) were added, and 1 mL of *p*-nitrophenyl phosphate solution was added to the other set of samples. After swirling both flasks for a few seconds to mix the contents, these were placed in an incubator at 37 °C for 1 h. Calcium chloride (1 mL of 0.5 M) and 4 mL of 0.5 M of NaOH were added after incubation. Flasks were swirled for a few seconds, and 1 mL of *p*-nitrophenyl phosphate solution was added to the remaining set of samples. All suspensions were filtered through Whatman No. 1 filter paper quickly, and the yellow color intensity was measured at a 440 nm wavelength.

The fluorescein diacetate (FDA) hydrolytic activity was determined by the method of Green et al. [54]. A total of 0.5 mg of soil was added to 25 mL of sodium phosphate (0.06 M; pH = 7.6). To all assay vials, 0.25 mL of 4.9 mM of FDA substrate solution was added. All vials were vortexed and incubated in a water bath at 37 °C for 2 h. Then, soil suspension was centrifuged at 8000 rpm for 5 min. The clear supernatant was measured at 490 nm against a reagent blank solution using a spectrophotometer.

Dehydrogenase activity (DHA) was determined using the method described by Casida Jr. et al. [55]. Fresh homogenized soil samples (5 g) were placed in test tubes, and 5 ml of substrate 3% *v*/*w* 2,3,5-triphenyltetrazolium chloride (TTC) was added. The tubes were incubated at 25 °C for 24 h. A blank sample was similarly prepared, with 1 mL of a 3% TTC solution phosphate buffer being introduced. After incubation, the samples were centrifuged at 4500 rpm for 10 min. The supernatant liquid was discarded. The formed triphenyl-formazan (TPF) was extracted with methanol. To each tube, 5 mL of methanol were added, and then the tubes were vigorously shaken for a few min. The operation was repeated twice (10 mL of methanol was used for extraction). Again, the tubes were centrifuged. The obtained supernatant liquid was poured into a clean tube, and the absorbance of the solution was measured at 485 nm.

### 2.7. Statistical Analyses

Experimental data were analyzed with StatView Software using ANOVA. The significance of the effect of treatment was determined by the magnitude of the F value (*p* < 0.05 < 0.001).

## 3. Results

**Figure 1:**
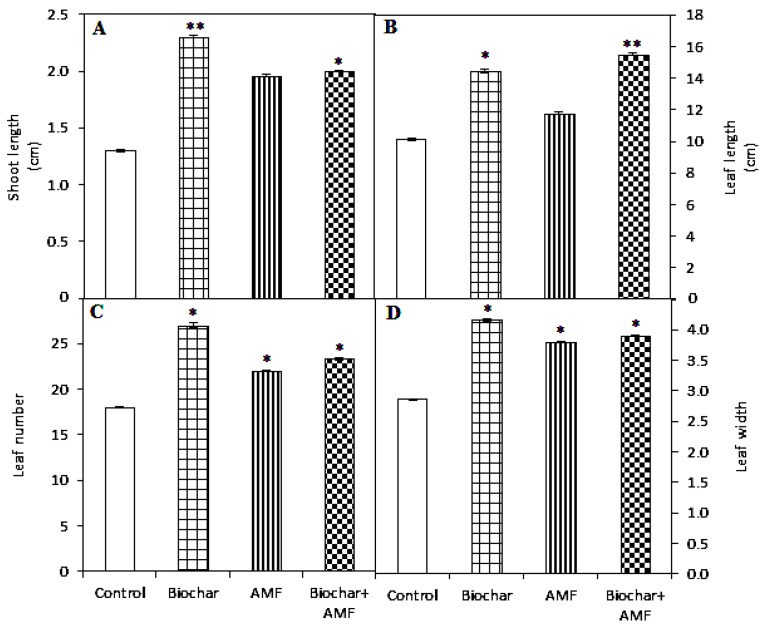
Biochar and AMF for the improvement of the shoot length (**A**), leaf length (**B**), leaf number (**C**), and leaf width (**D**) of spinach. Data are the means of three replicates (*n* = 3); * differed significantly at *p* < 0.05 *, *p* < 0.01 **.

AMF application significantly increased the leaf number and leaf width (Figure 1). Biochar treatment significantly increased the shoot length by 77%, leaf length by 43%, leaf number by 50%, and leaf width by 45% compared to the control. The combined biochar and AMF significantly increased the shoot length and leaf length by 54% and 53%, respectively, over the control. Similarly, the combined biochar and AMF had a positive effect on the leaf number and leaf width, with a 30% and 36% increase, respectively, compared to the control.

**Figure 2:**
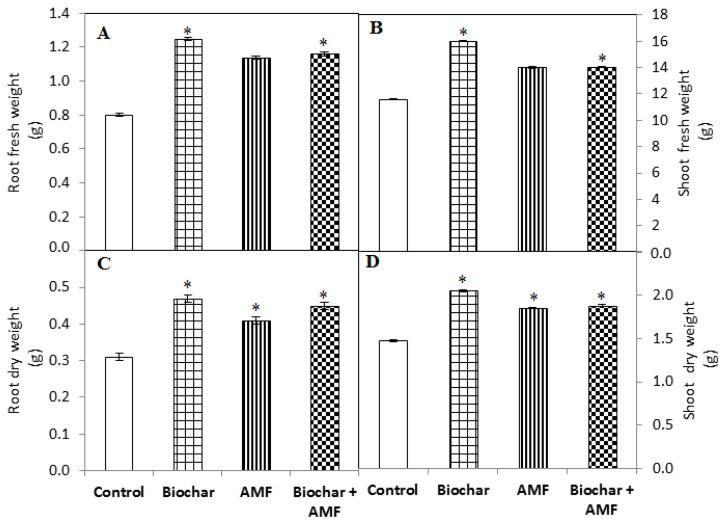
Biochar and AMF for the improvement of the root fresh weight (**A**), shoot fresh weight (**B**), root dry weight (**C**), and shoot dry weight (**D**) of spinach. Data are the means of three replicates (*n* = 3); * differed significantly at *p* < 0.05 *.

The highest root and shoot fresh weight and dry weight were recorded with the biochar treatment and combined biochar and AMF (Figure 2). The root fresh weight (56%) and the root dry weight (52%) were significantly improved by the biochar treatment compared to the control (Figure 2). The biochar treatment significantly enhanced the shoot fresh weight by 38% and the shoot dry weight by 39% over the control. AMF treatment slowly increased the shoot fresh weight and shoot dry weight. AMF treatment significantly increased the root and shoot dry weight compared to the control. The combined application of biochar and AMF significantly enhanced the root fresh weight and dry weight by 45% and 45%, respectively, compared to the control. The shoot fresh weight (27%) and dry weight (28%) were also significantly enhanced by the combined application of biochar and AMF compared to the control.

**Figure 3:**
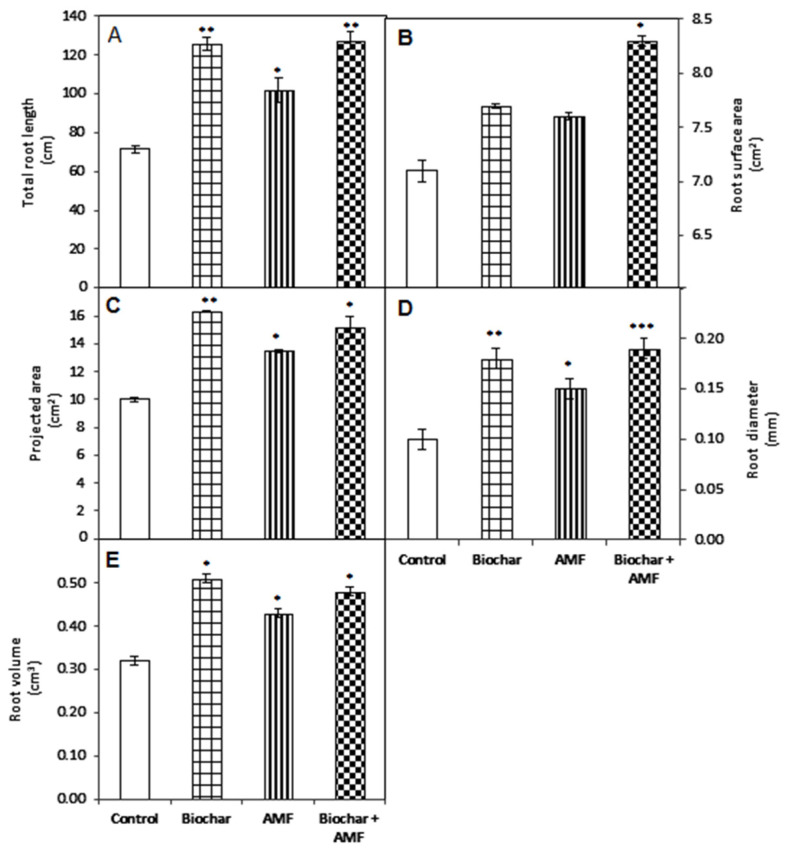
Biochar and AMF for the improvement of the root morphological traits of spinach. (**A**) Root length, (**B**) Root Surface are, (**C**) Root projected Area, (**D**) Root Diameter, (**E**) Root Volume. Data are the means of three replicates (*n* = 3); * differed significantly at *p* < 0.05 *, *p* < 0.01 **, *p* < 0.001 ***.

AMF treatment enhanced the total root length, projected area, root diameter, and root volume by 43%, 35%, 50%, and 34%, respectively, compared to the control (Figure 3). Biochar treatment significantly increased the projected area and root volume by 63% and 59%, respectively, compared to the control. The total root length and root diameter were significantly enhanced by 76% and 80%, respectively, by the biochar treatment compared to the control. The combined biochar and AMF treatment significantly increased the total root length by 78% and root diameter by 90% over the control. The projected area and root volume were increased by 52% and 50%, respectively, by the combined biochar and AMF.

**Figure 4:**
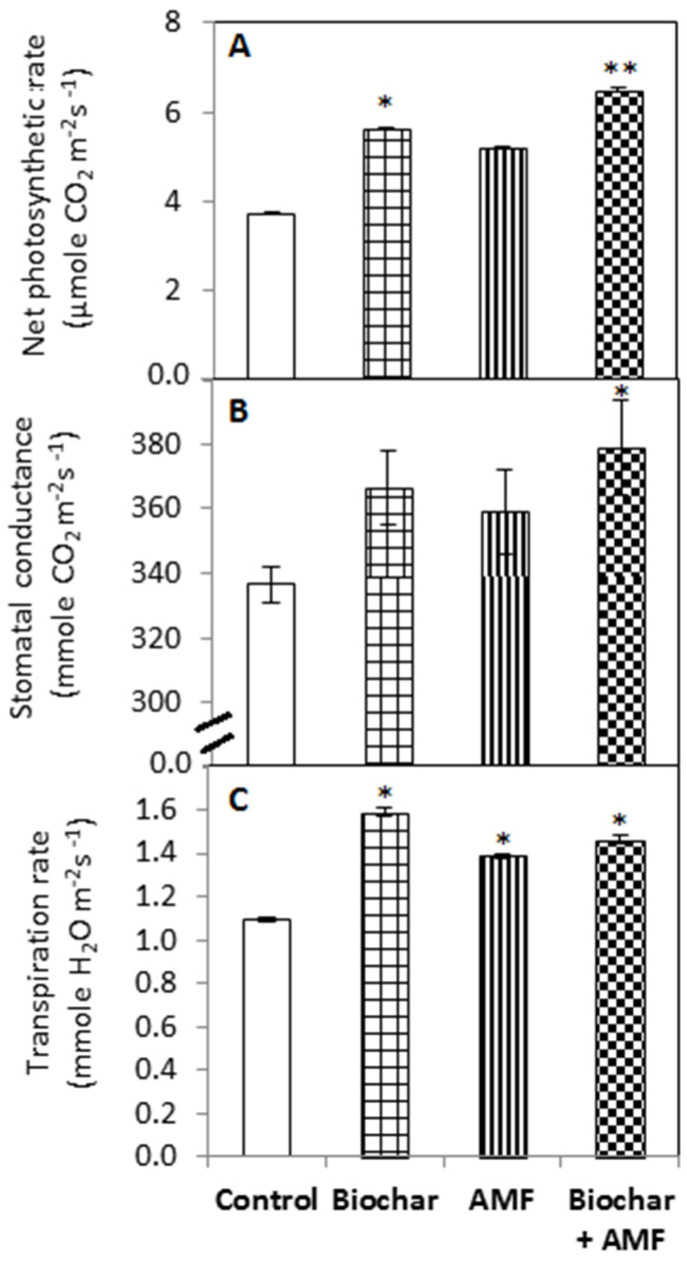
Biochar and AMF for the improvement of photosynthetic parameters. (**A**) Net photosynthesis, (**B**) Stomatal Conductance, (**C**) Transpiration rate. Data are the means of three replicates (*n* = 3); * differed significantly at *p* < 0.05 *, *p* < 0.01 **.

The biochar alone and the combined biochar and AMF significantly increased the photosynthetic rate by 50% and 74%, respectively, compared to the control (Figure 4). The stomatal conductance was significantly increased by the combined biochar and AMF compared to the control. The transpiration rate was significantly increased by all of the treatments compared with the control; the highest rate was recorded with the biochar treatment. Biochar treatment alone significantly enhanced the transpiration rate by 45% compared to the control.

**Figure 5:**
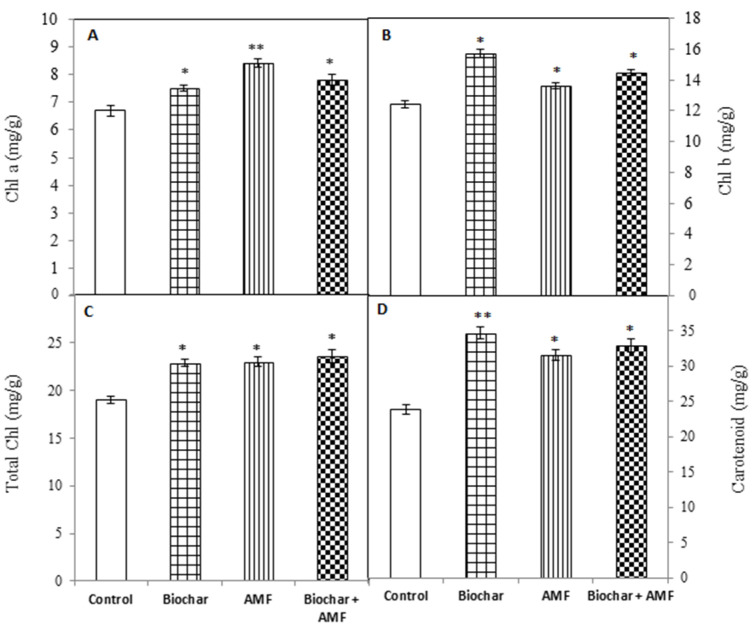
Biochar and AMF for the improvement of the photosynthetic pigment content of leaf (**A**) chlorophyll a (Chl a) content, (**B**) chlorophyll b (Chl b) content, (**C**) total chlorophyll content, and (**D**) carotenoid content. Data are the means of three replicates (*n* = 3); * differed significantly at *p* < 0.05 *, *p* < 0.01 **.

All of the treatments improved the photosynthetic pigment content of the leaf compared to the control (Figure 5). The biochar significantly increased the content of total chlorophyll, chlorophyll a and b, and carotenoid of the leaf by 20%, 12%, 27%, and 46%, respectively, over the control (Figure 5). The AMF alone significantly increased the total chlorophyll content, chlorophyll a and b content, and carotenoid content of the leaf by 21%, 25%, 10%, and 33%, respectively. The combined treatment with biochar and AMF significantly increased the total chlorophyll content, chlorophyll a and b content, and carotenoid content of the leaf by 24%, 16%, 17%, and 38%, respectively, over the control.

**Figure 6:**
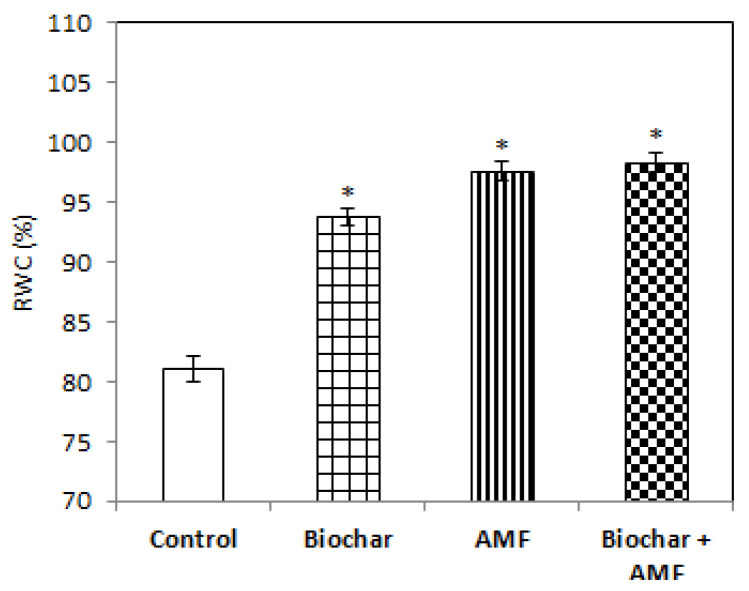
Biochar and AMF for improvement of the relative water content of the leaf. Data are the means of three replicates (*n* = 3); * differed significantly at *p* < 0.05 *.

All of the treatments, that is, the biochar alone, AMF alone, and biochar and AMF combined, increased the relative water content of the leaf compared to the control (Figure 6). The highest relative water content of the leaf was recorded for the combined biochar and AMF treatment; the content was 21% higher than with the control. In the treatment with biochar alone or AMF alone, there was an increase in the relative water content of 15% and 20%, respectively, compared to the control.

**Figure 7:**
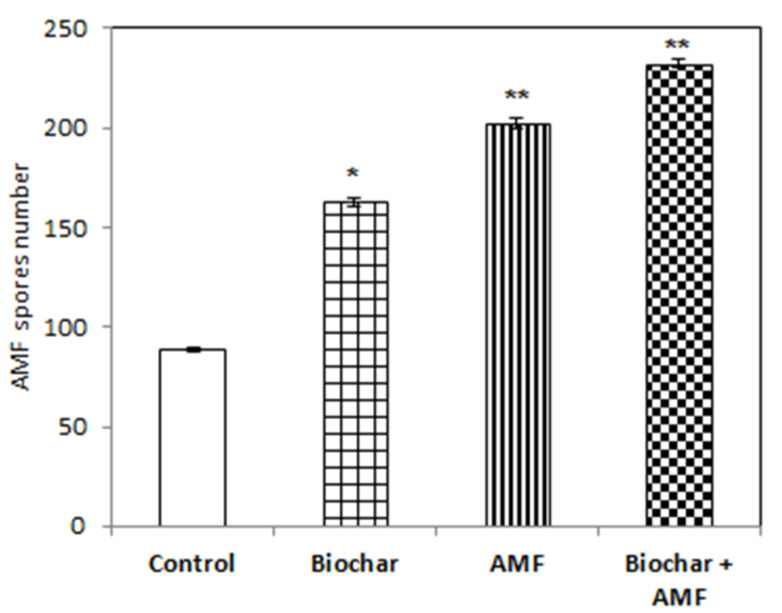
Biochar and AMF for the improvement of the AMF spores in soil. Data are the means of three replicates (*n* = 3); * differed significantly at *p* < 0.05 *, *p* < 0.01 **.

Both the treatment with AMF alone and the treatment with AMF and biochar were more effective in increasing the AMF spores in soil than the control (Figure 7). The AMF spores in soil increased by 126% to 150% with the AMF alone and with the combined biochar and AMF compared to the control. There was an 82% increase in AMF spores in soil with the biochar treatment over the control.

**Figure 8:**
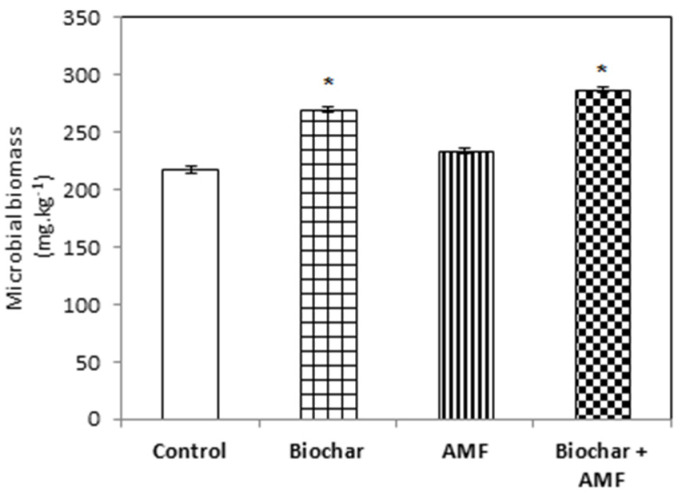
Biochar and AMF for the improvement of the microbial biomass in soil. Data are the means of three replicates (*n* = 3), * asterisk differed significantly at *p* < 0.05 *.

Both the treatment with biochar alone and the combined biochar and AMF treatment increased the carbon in the microbial biomass in soil compared to the control (Figure 8). The highest level of carbon was recorded with the combined biochar and AMF treatment compared with all other treatments; the level was 32% higher than with the control.

**Figure 9:**
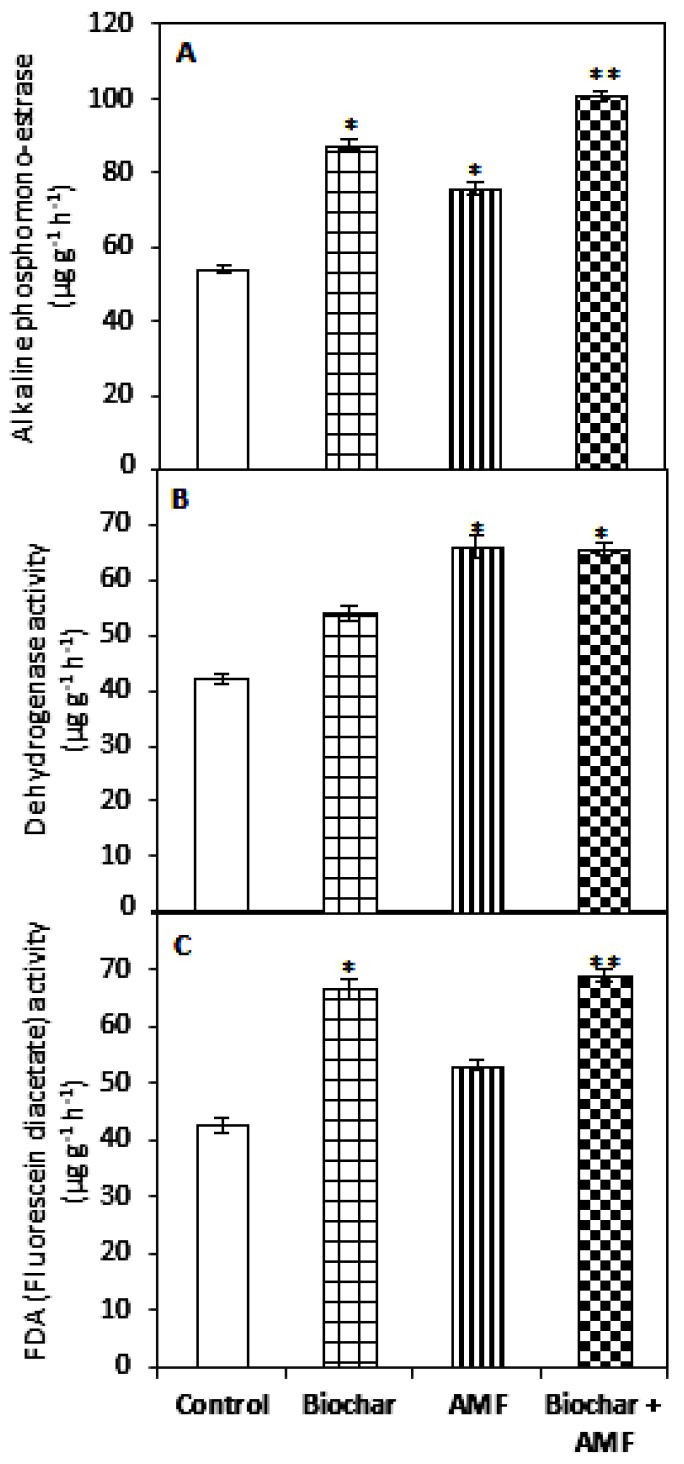
Biochar and AMF for the improvement of soil enzymes. (**A**) Alkaline phosphomonoester esterase, (**B**) Dehydrogenase, (**C**) Fluorescein diacetate activity. Data are the means of three replicates (*n* = 3); * differed significantly at *p* < 0.05 *, *p* < 0.01 **.

All of the treatments had a positive effect on the activity of alkaline phosphomonoestrase and increased the enzymatic activity in different treatments; the range was 62% to 86% (Figure 9). The highest alkaline phosphomonoestrase acitivity in soil was recorded for the combined biochar and AMF treatment. The treatment, AMF alone (57%), and the treatment with biochar and AMF (55%) had a positive effect on the dehydrogenase activity of soil compared to the control. Furthermore, biochar and the treatment with combined biochar and AMF had a beneficial effect on the fluorescein diacetate activity of soil compared with the other treatments. The highest increase in the fluorescein diacetate activity was recorded for the combined biochar and AMF; it was 62% higher than with the control.

## 4. Discussion

### 4.1. Effect of Biochar and AMF on Growth of Spinach Plants

In general, biochar treatment promoted attributes of plant growth, namely, the shoot length, leaf length, leaf number, and leaf width, which were significantly higher compared to the control. Similarly, the biochar treatment significantly increased the root and shoot fresh weight and root and shoot dry weights compared to the control. This finding is consistent with the report of Bu et al. [56], who observed a significant enhancement in the growth of *Robinia pseudoacacia* L. with biochar. Similarly, Hilioti et al. [57] reported that castor stalk biochar enhanced the growth of the castor plant. Numerous researchers have reported that biochar increased the plant growth and yield in different crops [7,23,24,25,58]. The positive effect of biochar amendment on the root length, shoot length, root biomass, shoot biomass, and yield in French beans was noted by Saxena et al. [20]. Similarly, Carter et al. [27] observed that the application of rice husk biochar increased the final biomass, root biomass, plant height, and number of leaves of lettuce (*Lactuca sativa*) and cabbage (*Brassica chinensis*) compared to plants that did not receive the biochar treatment. Enhanced dry weight, leaf biomass, and root biomass with biochar applications were also reported by Trupiano et al. [19]. Similarly, rice straw biochar significantly promoted the plant height, number of bolls per plant, average boll weight, and seed cotton yield compared with the control treatment, as reported by Qayyum et al. [16].

There was a significant increase in the leaf number, leaf width, shoot fresh weight, and shoot and root dry weight with AMF treatment. Many studies have reported that the application of AMF increased plant growth parameters [59,60,61,62]. Sharma and Kayang [63] reported that the application of AMF noticeably increased plant growth parameters of tea (*Camellia sinensis* L.), such as the number of leaves, leaf area, plant height, shoot length, root length, and root and shoot weight. The combined application of biochar and AMF had a positive effect on the leaf number, leaf length, and shoot and root fresh and dry weights compared to the control. A study by Li and Cai [64] found that biochar and AMF both improved the growth performance of maize. Similar results were reported by Budi and Setyaningsih [65]; their study showed that biochar and AMF significantly increased the plant height, diameter, shoot dry weight, and root dry weight compared to the control plant.

### 4.2. The Effect of Biochar and AMF on Root Morphological Traits

Biochar treatment clearly resulted in improved root morphological parameters, such as the total root length, projected area, root diameter, and root volume, compared to the control. Several studies have reported that biochar application improved plant roots [18,22,66], thereby confirming our results. Bu et al. [56] reported a significant increase in the root length, root surface area, and root volume following the application of rice husk biochar and woodchip biochar. Similar results of significant improvement in root growth due to the addition of biochar were also reported by Trupiano et al. [19]. Zhang et al. [67] found that biochar addition increased the taproot length, root volume, and total root absorption area in tobacco. Li and Cai [64] indicated that the addition of biochar significantly altered the root morphology under both 40% FWC and 60% FWC.

There was a significant enhancement of the total root length, projected area, root diameter, and root volume with AMF treatment compared to the control. In tomato seedlings inoculated with AMF, there was an increase in the total root length and number of root tips [68]. Bi et al. [69] found that AMF could alleviate root damage stress by changing the root morphology. *Melia azedarach* inoculated with *Gigaspora margarita* had a significantly higher plant height, diameter, and shoot and root dry weight [65]. Data regarding the combined biochar and AMF treatment showed a significantly increased total root length and root diameter compared with all of the other treatments. This finding confirms an earlier report by Hashem et al. [48] that found that combined biochar and AMF treatment significantly increased the root length of the chickpea. Biochar in combination with AMF plays a key role in the plant utilization of underground water and nutrients, and this is also dependent on the root architecture [70].

### 4.3. Effect of Biochar and AMF on Plant Physiological Properties

The study showed that the addition of biochar had a positive effect on the physiological properties of spinach. The net photosynthesis rate and transpiration rate were significantly increased by biochar treatment alone. Biochar treatment also significantly increased the content of chlorophyll a, chlorophyll b, total chlorophyll, carotenoid, and relative water of the leaf over the control. Many researchers found that biochar application increased the photosynthesis, chlorophyll content, and transpiration rate in different plants [19,28,29,71]. He et al. [72] reported that the application of biochar significantly increased the photosynthesis rate and chlorophyll concentration in C_3_ plants. Sarma et al. [25] found a strong positive effect of biochar amendment on the photosynthesis rate in okra. Hashem et al. [48] indicated that the application of biochar enhanced the amount of chlorophyll a, chlorophyll b, and total photosynthetic pigments.

**Figure 10:**
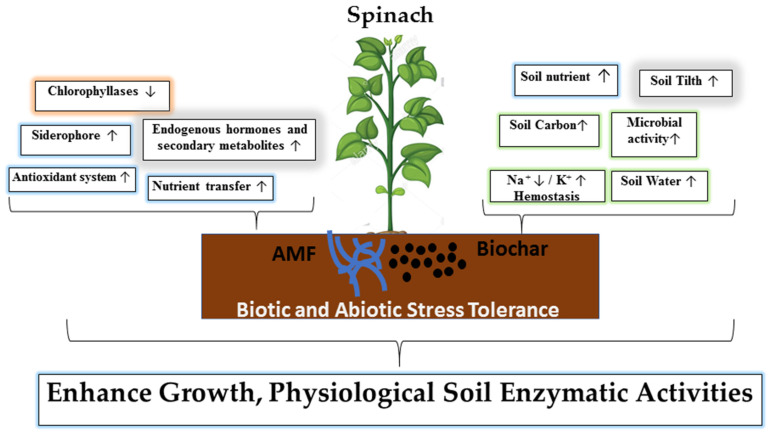
Summary for the action mechanism for the combined effect of biochar and AMF for the improvement of plant growth and soil enzymatic activity.

The net photosynthetic rate, stomatal conductance, and transpiration rate were increased by treatment with AMF alone. AMF alone significantly increased the content of chlorophyll a, chlorophyll b, total chlorophyll, carotenoid, and relative water of the leaf. Similar results have been reported by Ren et al. [73] which showed that inoculation with AMF significantly improved the antioxidant enzymatic activity and net photosynthesis rate in Zea mays. AMF inoculation increased the chlorophyll content and photosynthesis rate of maize and chickpeas [48,64]. The combined application of biochar and AMF had a positive effect on the net photosynthesis rate, stomatal conductance, transpiration rate, relative water content, and photosynthetic pigments compared to the control (Figure 4, Figure 5 and Figure 6). Similar results have been reported by Hashem et al. [48], showing that the combined application of AMF and biochar significantly increased the photosynthetic rate, relative water content, chlorophyll a, chlorophyll b and total chlorophylls in chickpea under normal condition. Similar findings confirming the significantly enhancement of the chlorophyll content and photosynthetic rate in maize were reported by Li and Cai [64]. As shown in Figure 10, they suggest that AMF and biochar increase nitrogen fixation and siderophore production, as well as enhance nutrient availability and absorption. Moreover, they trigger endogenous phytohormone synthesis and antioxidant production.

### 4.4. Effect of Biochar and AMF on AMF Spore Number, Microbial Biomass, and Soil Enzymatic Activity

Biochar treatment significantly increased the alkaline phosphomonoestrase activity and fluorescein diacetate activity compared with the control. Similar findings confirming enhanced soil enzymatic activity due to the addition of soybean biochar were reported by Jabborova et al. [18]. Bailey et al. [74] and Ma et al. [24] also observed increased enzymatic activity of soil due to biochar application. Several studies have reported an increase in the enzymes protease, chymotrypsin, trypsin, phosphohydrolase, lipase-esterase, and esterase with the application of biochar [17,19,75]. Oladele [76] reported significantly increased urease activity, invertase activity, and phosphatase activity with the application of biochar; the highest rate (12 t ha^−1^) was at soil depths of 0 to 0.1 m. The microbial biomass and the AMF spores in soil also increased compared to the control. A similar increase in AMF spores due to biochar application was reported by Hashem et al. [48]. Numerous studies have shown that biochar application promoted AMF colonization rates [77,78,79].

AMF treatment alone increased alkaline phosphomonoestrase activity, dehydrogenase activity, microbial biomass, and AMF spores compared to the control. Similar findings confirming that biochar increased urease and phosphatase activity and the soil microbial biomass were reported by Zhaoxiang et al. [26] and Li and Cai [64]. The combined application of biochar and AMF significantly increased the activity of alkaline phosphomonoestrase, dehydrogenase, and fluorescein diacetate, as well as the microbial biomass and AMF spores. Similar results have been reported by Li and Cai [64], who found that the combined application of AMF and biochar significantly improved the soil microbial activity in the maize rhizosphere. The mechanism of the combined effect of AMF and biochar is summarized in Figure 10.

## 5. Conclusions

Biochar application has facilitated the improvement of root morphological traits and plant growth. It also positively influences soil enzymatic activity. The combined application of biochar and AMF had a significantly positive impact on spinach plant growth, root morphological traits, physiological properties, and soil enzymatic activities. We conclude that further work on specific interactions between biochars has the potential to decrease the application of mineral fertilizers. The combined application of biochar and AMF can be used as an efficient biofertilizer to promote the plant growth and yield of spinach in field conditions.

## Data Availability

Not applicable.
